# Profiling of Gene Expression Biomarkers as a Classifier of Methotrexate Nonresponse in Patients With Rheumatoid Arthritis

**DOI:** 10.1002/art.40810

**Published:** 2019-03-19

**Authors:** Darren Plant, Mateusz Maciejewski, Samantha Smith, Nisha Nair, the Maximising Therapeutic Utility in Rheumatoid Arthritis Consortium, the RAMS Study Group, Kimme Hyrich, Daniel Ziemek, Anne Barton, Suzanne Verstappen

**Affiliations:** ^1^ Manchester University NHS Foundation Trust Manchester UK; ^2^ Pfizer Cambridge Massachusetts; ^3^ University of Manchester Manchester UK

## Abstract

**Objective:**

Approximately 30–40% of rheumatoid arthritis (RA) patients who are initially started on low‐dose methotrexate (MTX) will not benefit from the treatment. To date, no reliable biomarkers of MTX inefficacy in RA have been identified. The aim of this study was to analyze whole blood samples from RA patients at 2 time points (pretreatment and 4 weeks following initiation of MTX), to identify gene expression biomarkers of the MTX response.

**Methods:**

RA patients who were about to commence treatment with MTX were selected from the Rheumatoid Arthritis Medication Study. Using European League Against Rheumatism (EULAR) response criteria, 42 patients were categorized as good responders and 43 as nonresponders at 6 months following the initation of MTX treatment. Data on whole blood transcript expression were generated, and supervised machine learning methods were used to predict a EULAR nonresponse. Models in which transcript levels were included were compared to models in which clinical covariates alone (e.g., baseline disease activity, sex) were included. Gene network and ontology analysis was also performed.

**Results:**

Based on the ratio of transcript values (i.e., the difference in log_2_‐transformed expression values between 4 weeks of treatment and pretreatment), a highly predictive classifier of MTX nonresponse was developed using L2‐regularized logistic regression (mean ± SEM area under the receiver operating characteristic [ROC] curve [AUC] 0.78 ± 0.11). This classifier was superior to models that included clinical covariates (ROC AUC 0.63 ± 0.06). Pathway analysis of gene networks revealed significant overrepresentation of type I interferon signaling pathway genes in nonresponders at pretreatment (*P* = 2.8 × 10^−25^) and at 4 weeks after treatment initiation (*P* = 4.9 × 10^−28^).

**Conclusion:**

Testing for changes in gene expression between pretreatment and 4 weeks post–treatment initiation may provide an early classifier of the MTX treatment response in RA patients who are unlikely to benefit from MTX over 6 months. Such patients should, therefore, have their treatment escalated more rapidly, which would thus potentially impact treatment pathways. These findings emphasize the importance of a role for early treatment biomarker monitoring in RA patients started on MTX.

## Introduction

Low‐dose methotrexate (MTX) is the key therapy for the majority of patients with rheumatoid arthritis (RA). However, in ~30–40% of patients treated with MTX, disease activity is not adequately controlled 1, but current guidelines suggest that MTX treatment be administered for 6 months before a decision is made as to its efficacy 2. It is now well‐established that early, effective therapy prevents long‐term joint damage and disability 3, and the availability of biologic agents emphasizes the importance of identifying those patients who will not do well with MTX therapy, and who should, therefore, be fast‐tracked to more targeted therapies in order to protect against progressive and irreversible joint damage.

Although MTX has been used for more than 2 decades to treat RA, our ability to predict who will experience a good response versus nonresponse remains very limited. Clinical and demographic factors are only moderately predictive of the clinical response to MTX. For example, age and seropositivity (e.g., seropositive for rheumatoid factor, anti–cyclic citrullinated peptide antibodies [ACPAs]) are not robustly associated with MTX response 4, 5, 6, whereas male patients tend to respond better than female patients 7. Furthermore, patients with low levels of disease activity tend to respond better than those with higher disease activity. Finally, patients who take nonsteroidal antiinflammatory drugs tend to respond better to MTX than those who do not 7, while prior treatment with disease‐modifying antirheumatic drugs has been associated with MTX nonresponse 8.

Several studies have tested whether genetic and genomic factors can predict the response to MTX 9. However, many of the published studies have been small and have assessed a limited number of genes, with limited coverage. Moreover, the results of those studies have not been validated.

Expression microarrays have been investigated as a potential source of biomarkers that may be predictive of the treatment response in RA 10. The majority of studies have focused on response to biologic therapies, and not MTX 11, 12, and there has been little consistency in the findings. These inconsistencies could be attributable to differences in study design and inclusion criteria, the time point assessed, the sample sizes investigated, lack of appropriate model validation, the drugs studied, and the assessment of individual, rather than combined, groups of related transcripts 10. Nonetheless, in other diseases, gene expression has been used to stratify the underlying disease into subgroups with differing responses to treatments. For example, several markers that can predict the responsiveness to endocrine therapies in patients with breast cancer have been identified 13, 14, 15, 16, 17.

Therefore, the aim of the current study was to identify gene transcripts in patients with recent‐onset RA that could potentially be used to classify nonresponse to MTX at 6 months following the initiation of treatment, when tested either before MTX is initiated or at a time point (4 weeks) shortly after treatment initiation.

## Patients and methods

### Patients and samples

Patients in this study were participants in the Rheumatoid Arthritis Medication Study (RAMS), a national multicenter, longitudinal observational study in the UK that recruits patients with RA who have commenced MTX monotherapy for the first time. MTX was prescribed according to local practice. Patients were seen by a research nurse prior to commencement of MTX and at 3, 6, and 12 months thereafter. Clinical assessments included 28‐joint counts of swollen and tender joints. Patients completed health status questionnaires, including a self‐report of current functional disability using the Health Assessment Questionnaire (HAQ) (a score of ≤1 was considered low) 18. Blood samples were obtained at each visit, and serum was stored at −80°C prior to measuring the C‐reactive protein (CRP) level and ACPAs. The Disease Activity Score in 28 joints using CRP level (DAS28‐CRP) was calculated at baseline and at 6 months, and established European League Against Rheumatism (EULAR) response criteria were applied 19 to categorize patients as either MTX good responders or MTX nonresponders over the course of 6 months of treatment.

Samples of whole blood from the patients was drawn into Tempus blood tubes at the pretreatment and 4‐week time points, before being shipped to the central processing laboratory at the Arthritis Research UK Centre for Genetics and Genomics. The samples were logged onto a laboratory information management system and stored at −80°C.

### Expression profiling

Total RNA was extracted using a Tempus Spin RNA isolation kit, according to the manufacturer's protocol. After extraction, RNA was quantified using a Thermo Scientific Nanodrop ND‐1000 spectrophotometer, and RNA integrity was assessed using an Agilent Technologies 2100 Bioanalyzer. An optical density at 260/280 nm (OD_260/280 nm_) of ~2 and an OD_260/230 nm_ of 2–2.2 suggests that no contaminants were present within a sample, and an RNA integrity number of >6 was deemed to indicate sufficient RNA quality.

RNA samples were labeled with biotin and amplified using an Illumina TotalPrep RNA amplification kit. Following labeling and amplification, the RNA was re‐quantified and 750 ng was hybridized onto Illumina HumanHT‐12‐v4 Expression BeadChips (which target 47,000 probes), in accordance with the direct hybridization protocol. Scanning was performed using an Illumina iScan system, in order to collect raw intensity data from the expression BeadChips prior to export into GenomeStudio for further analysis.

### Data quality control

GenomeStudio software was used to assess control probe summary statistics and summarize bead‐level expression data. Quality control was performed using the limma Bioconductor package 20. Probes not expressed on any array or probe sequences with undesirable properties (e.g., poor mapping) were removed, and data were quantile normalized and log_2_ transformed. Potential batch effects were assessed by visual inspection of multidimensional scaling plots, and principal components analysis and hierarchical clustering of samples was performed to identify sample outliers.

### Statistical analysis

#### Classifier performance

We built statistical machine learning models to distinguish therapeutic nonresponders from responders (assessed at 6 months) using gene expression data at pretreatment and 4 weeks, and using the ratio of gene expression (i.e., the difference in log_2_‐transformed transcript expression intensity between 4 weeks of treatment and pretreatment [a total of 6 contrasts]). In addition, we built models based on clinical variables at pretreatment and 3 month. These models included sex, age at disease onset, HAQ score, smoking habits, ACPA positivity (titer >10 units/ml), number of swollen joints, number of tender joints, CRP levels, and patient's assessment of overall well‐being (on 100‐mm visual analog scale [VAS]).

For each contrast, we employed 3 state‐of‐the‐art machine learning methods with different characteristics: a linear method (regularized logistic regression), a nonlinear method (random forest), and, in the case of the contrasts using gene expression data, a pathway‐supported approach 21. Standardization was applied to all input data, and each method was run under a 10‐fold nested cross‐validation scheme (where hyperparameters were computed in each of the strata using an inner 5‐fold cross‐validation loop) to give accurate estimates of predicted performance. The performance of resulting models was reported using balanced accuracy and receiver operating characteristic (ROC) curves. Balanced accuracy and area under the ROC curves (AUCs) are reported as the mean ± SEM. To estimate feature importance, we averaged the model regression coefficients (mean ± SD) from across the cross‐validation runs.

#### Weighted genetic coexpression network analysis (WGCNA)

For modular analysis by WGCNA (details on the workflow are provided in ref. 22), we first calculated Pearson's correlations between all genes present in the data set. Next, an adjacency matrix was calculated by raising the absolute values of the correlation matrix to a power β, to penalize weak correlations and preserve stronger ones. The β value for the soft thresholding was chosen in each data set using the “scale‐free topology criterion.” Topologic overlap was then calculated to quantify gene coexpression relationships, considering each pair of genes in relation to all of the other genes in the coexpression network.

Hierarchical clustering was then used to construct a dendrogram with branches corresponding to genes within modules, determined using a dynamic tree‐cutting approach 22. For visualization, gene modules were given arbitrary color labels. Genes that were unassigned (i.e., not coexpressed) during network construction were arbitrarily labeled with a grey color. Gene counts in the intersection of corresponding modules between nonresponders and the consensus group of good responders and nonresponders at pretreatment and at 4 weeks were compared using the hypergeometric test *P* value for the overlap of the 2 modules.

In order to identify hub genes from the gene modules, an adjacency matrix was constructed for each gene, and connectivity was calculated as the sum of the adjacency to all other genes. Genes were then ranked by connectivity, and the top 20% of genes were selected from each module, the rationale being that only a fraction of genes in modules are likely to relate to the main biologic function 23.

### Pathway analysis

Functional analysis of hub genes derived from the modules were analyzed by hypergeometric testing on gene ontology terms 24. In addition, previous evidence of coexpression was investigated using data from Gene Expression Omnibus 25, 26.

## Results

### Samples

Following application of data quality control, 22,771 probes were identified and available for analysis at the 2 time points in samples of whole blood from 82 RA patients. The patients were categorized as either good responders (n = 42) or poor responders/nonresponders (n = 43) following 6 months of treatment with MTX. The pretreatment demographic and clinical characteristics of the patients are shown in Table 1.

**Table 1:** Pretreatment characteristics of the patients with rheumatoid arthritis^a^

Characteristic	EULAR good responders (n = 42)	EULAR nonresponders (n = 43)	*P*
Female, no. (%)	32 (76)	33 (77)	0.95
Age at onset, mean ± SD years	59 ± 15	55 ± 14	0.28
HAQ score, median (IQR)	1.18 (0.9–1.7)	1.0 (0.3–1.6)	0.07
MTX start dose, median (IQR) mg	12.5 (10–15)	10 (10–15)	0.87
Taking oral steroids, no. (%)	5 (12)	12 (27)	0.07
Disease duration, median (IQR) months	9.1 (4.2–15.3)	5.8 (3.1–21.7)	0.94
Smoking habits, no. never/past/current	23/11/8	16/17/10	0.25
ACPA positive, no. (%)	27 (64)	25 (58)	0.56
DAS28, mean ± SD	4.8 ± 1.0	4.0 ± 1.3	0.001
CRP, mean ± SD mg/liter	2.2 ± 1	1.7 ± 1	0.01
28‐joint swollen joint count, median (IQR)	5 (3–11)	3 (2–8)	0.02
28‐joint tender joint count, median (IQR)	8 (6–15)	6 (1–13)	0.05
Patient's assessment of overall well‐being, median (IQR) VAS score	44 (25–64)	32 (15–59)	0.07

a EULAR = European League Against Rheumatism; HAQ = Health Assessment Questionnaire; IQR = interquartile range; MTX = methotrexate; ACPA = anti–cyclic citrullinated peptide antibody; DAS28 = Disease Activity Score in 28 joints; CRP = C‐reactive protein; VAS = 100‐mm visual analog scale.

*P* values were derived by *t*‐test, Mann‐Whitney U test, and chi‐square test for comparisons of variables expressed as the mean, median, and number (%), respectively.

### Classifier performance

**Figure 1:**
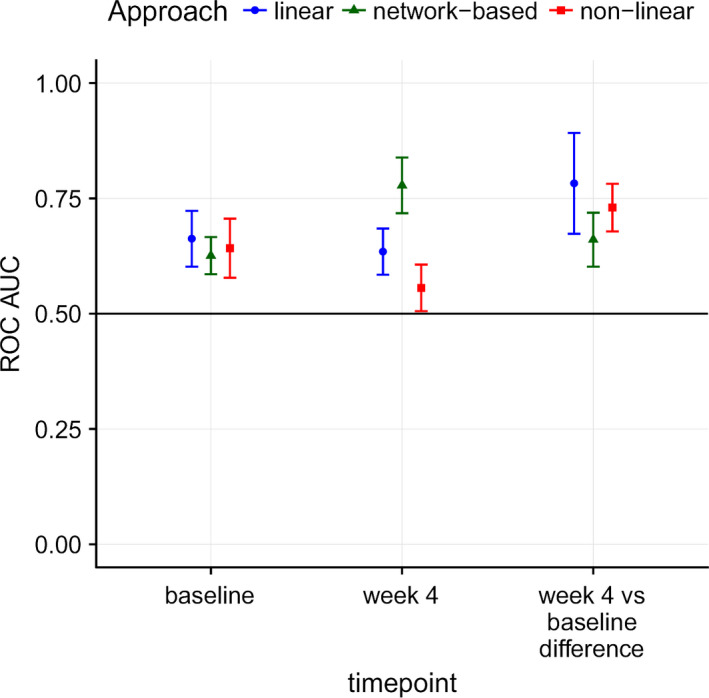
Performance of statistical machine learning models to distinguish therapeutic nonresponders from responders (assessed at 6 months) using gene expression data at pretreatment and 4 weeks after treatment initiation, and using the ratio of gene expression (i.e., the difference in log_2_‐transformed transcript expression intensity between 4 weeks of treatment and pretreatment). Area under the receiver operating characteristic (ROC) curves (AUCs) were calculated for estimating the predicted performance of a linear method (regularized logistic regression), a nonlinear method (random forest), and a network‐based approach to evaluating methotrexate nonresponse in patients with rheumatoid arthritis. Results are the mean ± SEM.

**Figure 2:**
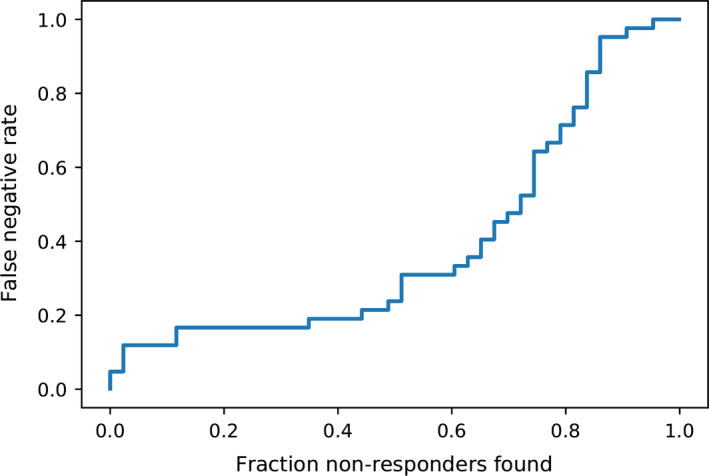
L2‐regularized logistic regression model performance using the gene expression ratio between 4 weeks and pretreatment. Results are the fraction of true nonresponders found versus false negative rate.

In the models based on transcriptomics data, a high level of prediction of MTX nonresponse was observed with the L2‐regularized logistic regression (linear method) model of the gene expression ratio between 4 weeks and pretreatment (Figures 1 and 2). Using this model, the balanced accuracy was a mean ± SEM 0.61 ± 0.10, and the ROC AUC was 0.78 ± 0.11. A very limited predictive utility was observed at the pretreatment time point. In contrast, the network‐based models had a good degree of predictive utility at the 4‐week time point (balanced accuracy 0.68 ± 0.06, ROC AUC 0.78 ± 0.06).

Very limited predictive utility was observed in the models based on the clinical data alone, either at baseline or at 3 months (e.g., with the linear method, at baseline, balanced accuracy 0.58 ± 0.5, ROC AUC 0.65 ± 0.06; at 3 months, balanced accuracy 0.62 ± 0.04, ROC AUC 0.70 ± 0.05) (see Supplementary Figure 1, available on the *Arthritis & Rheumatology* web site at http://onlinelibrary.wiley.com/doi/10.1002/art.40810/abstract). The transcripts with the largest positive impact on the performance of the models, based on the transcript ratio analysis, are presented in Supplementary Figure 2 (available on the *Arthritis & Rheumatology* web site at http://onlinelibrary.wiley.com/doi/10.1002/art.40810/abstract).

### Modular analysis using WGCNA

In samples of whole blood from RA patients at pretreatment, 9 modules (arbitrarily labeled in midnight blue, light green, royal blue, magenta, grey60, black, salmon, green‐yellow, and light‐yellow colors in Supplementary Figure 3, available on the *Arthritis & Rheumatology* web site at http://onlinelibrary.wiley.com/doi/10.1002/art.40810/abstract) were identified in nonresponders that had not been seen in pretreatment samples from the consensus group of good responders and nonresponders (i.e., labeled in grey [i.e., unassigned] in the consensus network; see Supplementary Figure 3).

In samples at 4 weeks following initiation of treatment with MTX, 4 modules (arbitrarily labeled in salmon, light cyan, grey60, and yellow colors in Supplementary Figure 4, available on the *Arthritis & Rheumatology* web site at http://onlinelibrary.wiley.com/doi/10.1002/art.40810/abstract) were identified in nonresponders that were unassigned (i.e., labeled in grey) in the consensus samples.

### Pathway analysis

Functional analysis of the gene lists derived from the identified modules (as described above) revealed a number of biologic processes relevant to inflammatory processes, such as genes involved in the response to type I interferon (*P* = 2.8 × 10^−25^) at pretreatment, and the type I interferon signaling pathway (*P* = 4.9 × 10^−28^) at 4 weeks (labeled in light green and light cyan, respectively; see Supplementary Tables 1, 2, and 3, available on the *Arthritis & Rheumatology* web site at http://onlinelibrary.wiley.com/doi/10.1002/art.40810/abstract). Using data from the Gene Expression Omnibus database, we found that >90% of hub genes from within these modules had prior evidence of coexpression, thus validating the gene network approach (see Supplementary Figures 5 and 6, available on the *Arthritis & Rheumatology* web site at http://onlinelibrary.wiley.com/doi/10.1002/art.40810/abstract).

We tested the interferon pathway transcripts for their predictive accuracy as a classifier of MTX nonresponse. Although we observed nonrandom performance (e.g., with the linear method at baseline, balanced accuracy 0.52 ± 0.04, ROC AUC 0.64 ± 0.06), this classifier performed less well than the model that included all transcripts present on the array (see Supplementary Figure 7, available on the *Arthritis & Rheumatology* web site at http://onlinelibrary.wiley.com/doi/10.1002/art.40810/abstract).

## Discussion

In this study, we performed gene expression profiling in samples of whole blood collected at pretreatment and 4 weeks following the initiation of MTX therapy from patients with RA who had been started on MTX for the first time. By assessing a number of cutting edge model‐building approaches, we developed a gene expression classifier that could potentially provide an early‐response biomarker of MTX inefficacy. The classifier was found to be stable in cross‐validation (SEM of 0.11 for the ROC AUC) and performed better than models that included the clinical covariates alone.

Pathway analysis revealed that genes involved in the response to type I interferon (*P* = 2.8 × 10^−25^) and the type I interferon signaling pathway (*P* = 4.9 × 10^−28^) were enriched in coexpressed gene modules identified in nonresponsive patients at pretreatment and at 4 weeks post–treatment initiation, respectively. Importantly, type I IFN signaling activates the JAK/STAT pathway and influences the development of innate and adaptive immune responses 27. Type I interferon gene responses are known to be increased in RA, to be correlated with autoantibody production 28, and to potentially be correlated with the response to tumor necrosis factor inhibitor therapy 28, 29, 30, 31, 32, 33, 34. It is important to note that the coexpressed genes were not differentially expressed between responder groups or between time points (data not shown).

The results of this study highlight the potential of early treatment biomarker monitoring in RA, and raise important questions regarding acceptable levels of performance for complementary diagnostic testing. To address this, decisions by key stakeholders (e.g., patient groups, clinicians) need to be made. Acceptable classifier performance must also be viewed in context, since ~46% of RA patients started on MTX therapy will discontinue the treatment by 3 years, due to intolerance/safety and inefficacy 35. Therefore, we believe that high recall (correctly identifying nonresponders) is preferable even at the expense of misclassifying a fraction of good responders. For example, the current model was able to detect ~50% of nonresponders at the expense of a false negative rate of ~20% (Figure 2).

The strengths of the current study include a large sample size, the availability of genome‐wide transcript data at pretreatment and also during early treatment, rigorous internal model validation, and a focus on one drug (i.e., MTX). Furthermore, a comparison with external data confirmed that the gene expression networks identified in the current data have previous evidence of coexpression, providing external validity.

A limitation in investigating MTX nonresponse is the semiquantitative nature of the methods used to approximate disease activity, for example, the DAS28 36 and related EULAR response classification. The DAS28 score is composed of both objective (e.g., swollen joint count) and subjective (e.g., tender joint count) measures. As a result of it being made up of several components, it can be difficult to interpret, particularly because the subjective measures receive more weighting in the score calculation, and tend to correlate more strongly with psychological variables, such as anxiety 37 and fibromyalgia tender points 38. Improved classifier performance might therefore be achieved if a biologic measure of disease activity, particularly one that might be more strongly reflective of synovitis levels, were to be used to assess the treatment response, as opposed to the DAS28 or its components.

Another potential limitation to the current study is the use of whole blood for transcript profiling. While the purpose of the study was not to resolve mechanisms of nonresponse, model performance may have been improved by targeting enriched cell subsets within the blood.

The utility of a gene expression classifier of MTX nonresponse will now require validation, not only in independent samples but also using independent technology. For example, more mileage may be gained from RNA sequencing, as opposed to array‐based data sets. If the predictive utility of gene expression data can be confirmed, this could pave the way for a paradigm shift in treatment outcomes from clinically based treat‐to‐target approaches to biologically driven precision medicine.

In conclusion, these data reveal a potential role for early treatment biomarker monitoring in RA patients started on MTX, and highlight the utility of machine learning and network‐based approaches in investigations of treatment response in inflammatory diseases.

## Author contributions

All authors were involved in drafting the article or revising it critically for important intellectual content, and all authors approved the final version to be published. Dr. Barton had full access to all of the data in the study and takes responsibility for the integrity of the data and the accuracy of the data analysis.

### Study conception and design

Plant, Maciejewski, Hyrich, Ziemek, Barton, Verstappen.

### Acquisition of data

Smith, Nair, Barton, Verstappen.

### Analysis and interpretation of data

Plant, Maciejewski, Hyrich, Ziemek, Barton, Verstappen.

## Role of the study sponsor

Pfizer provided support for the study but had no role in the study design or in the collection, analysis, or interpretation of the data, the writing of the manuscript, or the decision to submit the manuscript for publication. Publication of this article was not contingent upon approval by Pfizer.

## Supporting information

Supplementary Figures

Supplementary Tables

## References

[1] Salliot C , van der Heijde D . Long‐term safety of methotrexate monotherapy in patients with rheumatoid arthritis: a systematic literature research. Ann Rheum Dis 2009;68:1100–4. 19060002 10.1136/ard.2008.093690 PMC2689525

[2] National Insitute for Health and Care Excellence . Rheumatoid arthritis in adults: management. URL: https://www.nice.org.uk/guidance/cg79.

[3] Farragher TM , Lunt M , Fu B , Bunn D , Symmons DP . Early treatment with, and time receiving, first disease‐modifying antirheumatic drug predicts long‐term function in patients with inflammatory polyarthritis. Ann Rheum Dis 2010;69:689–95. 19858538 10.1136/ard.2009.108639 PMC2927614

[4] Bluett J , Sergeant J , MacGregor AJ , Symmons D , Verstappen S . Predictors of oral methotrexate failure in patients with early inflammatory arthritis: a competing risks analysis. Rheumatology (Oxford) 2015;54 Suppl 1:i71.

[5] Wessels JA , van der Kooij SM , le Cessie S , Kievit W , Barerra P , Allaart CF , et al. A clinical pharmacogenetic model to predict the efficacy of methotrexate monotherapy in recent‐onset rheumatoid arthritis. Arthritis Rheum 2007;56:1765–75. 17530705 10.1002/art.22640

[6] Rheumatoid Arthritis Clinical Trial Archive Group . The effect of age and renal function on the efficacy and toxicity of methotrexate in rheumatoid arthritis. J Rheumatol 1995;22:218–23. 7738941

[7] Hoekstra M , van Ede AE , Haagsma CJ , van de Laar MA , Huizinga TW , Kruijsen MW , et al. Factors associated with toxicity, final dose, and efficacy of methotrexate in patients with rheumatoid arthritis. Ann Rheum Dis 2003;62:423–6. 12695153 10.1136/ard.62.5.423 PMC1754533

[8] Lie E , van der Heijde D , Uhlig T , Heiberg MS , Koldingsnes W , Rødevand E , et al. Effectiveness and retention rates of methotrexate in psoriatic arthritis in comparison with methotrexate‐treated patients with rheumatoid arthritis. Ann Rheum Dis 2010;69:671–6. 19740904 10.1136/ard.2009.113308

[9] Plant D , Wilson AG , Barton A . Genetic and epigenetic predictors of responsiveness to treatment in RA. Nat Rev Rheumatol 2014;10:329–37. 24535543 10.1038/nrrheum.2014.16

[10] Smith SL , Plant D , Eyre S , Barton A . The potential use of expression profiling: implications for predicting treatment response in rheumatoid arthritis. Ann Rheum Dis 2013;72:1118–24. 23486412 10.1136/annrheumdis-2012-202743

[11] Hobl EL , Mader RM , Erlacher L , Duhm B , Mustak M , Bröll H , et al. The influence of methotrexate on the gene expression of the pro‐inflammatory cytokine IL‐12A in the therapy of rheumatoid arthritis. Clin Exp Rheumatol 2011;29:963–9. 22133036

[12] Parker A , Izmailova ES , Narang J , Badola S , Le T , Roubenoff R , et al. Peripheral blood expression of nuclear factor‐κb‐regulated genes is associated with rheumatoid arthritis disease activity and responds differentially to anti‐tumor necrosis factor‐α versus methotrexate. J Rheumatol 2007;34:1817–22. 17696278

[13] Dowsett M , Smith IE , Ebbs SR , Dixon JM , Skene A , A'Hern R , et al. Prognostic value of Ki67 expression after short‐term presurgical endocrine therapy for primary breast cancer. J Natl Cancer Inst 2007;99:167–70. 17228000 10.1093/jnci/djk020

[14] Dowsett M , Allred C , Knox J , Quinn E , Salter J , Wale C , et al. Relationship between quantitative estrogen and progesterone receptor expression and human epidermal growth factor receptor 2 (HER‐2) status with recurrence in the arimidex, tamoxifen, alone or in combination trial. J Clin Oncol 2008;26:1059–65. 18227529 10.1200/JCO.2007.12.9437

[15] Harvey JM , Clark GM , Osborne CK , Allred DC . Estrogen receptor status by immunohistochemistry is superior to the ligand‐binding assay for predicting response to adjuvant endocrine therapy in breast cancer. J Clin Oncol 1999;17:1474–81. 10334533 10.1200/JCO.1999.17.5.1474

[16] Houston SJ , Plunkett TA , Barnes DM , Smith P , Rubens RD , Miles DW . Overexpression of c‐erbB2 is an independent marker of resistance to endocrine therapy in advanced breast cancer. Br J Cancer 1999;79:1220–6. 10098763 10.1038/sj.bjc.6690196 PMC2362250

[17] McGuire WL . Steroid receptors in human breast cancer. Cancer Res 1978;38:4289–91. 698967

[18] Fries JF , Spitz PW , Young DY . The dimensions of health outcomes: the health assessment questionnaire, disability and pain scales. J Rheumatol 1982;9:789–93. 7175852

[19] DAS28 . EULAR response criteria. URL: https://www.das-score.nl/das28/en/difference-between-the-das-and-das28/importance-of-das28-and-tight-control/eular-response-criteria.html.

[20] Ritchie ME , Phipson B , Wu D , Hu Y , Law CW , Shi W , et al. Limma powers differential expression analyses for RNA‐sequencing and microarray studies. Nucleic Acids Res 2015;43:e47. 25605792 10.1093/nar/gkv007 PMC4402510

[21] Zarringhalam K , Degras D , Brockel C , Ziemek D . Robust phenotype prediction from gene expression data using differential shrinkage of co‐regulated genes. Sci Rep 2018;8:1237. 29352257 10.1038/s41598-018-19635-0 PMC5775343

[22] Langfelder P , Horvath S . WGCNA: an R package for weighted correlation network analysis. BMC Bioinformatics 2008;9:559. 19114008 10.1186/1471-2105-9-559 PMC2631488

[23] Van Dam S , Võsa U , van der Graaf A , Franke L , de Magalhães JP . Gene co‐expression analysis for functional classification and gene–disease predictions. Brief Bioinform 2018;19:575–92. 28077403 10.1093/bib/bbw139 PMC6054162

[24] Falcon S , Gentleman R . Using GOstats to test gene lists for GO term association. Bioinformatics 2007;23:257–8. 17098774 10.1093/bioinformatics/btl567

[25] Barrett T , Troup DB , Wilhite SE , Ledoux P , Rudnev D , Evangelista C , et al. NCBI GEO: archive for high‐throughput functional genomic data. Nucleic Acids Res 2009;37:D885–90. 18940857 10.1093/nar/gkn764 PMC2686538

[26] Warde‐Farley D , Donaldson SL , Comes O , Zuberi K , Badrawi R , Chao P , et al. The GeneMANIA prediction server: biological network integration for gene prioritization and predicting gene function. Nucleic Acids Res 2010;38:W214–20. 20576703 10.1093/nar/gkq537 PMC2896186

[27] Ivashkiv LB , Donlin LT . Regulation of type I interferon responses. Nat Rev Immunol 2014;14:36–49. 24362405 10.1038/nri3581 PMC4084561

[28] Castañeda‐Delgado JE , Bastián‐Hernandez Y , Macias‐Segura N , Santiago‐Algarra D , Castillo‐Ortiz JD , Alemán‐Navarro AL , et al. Type I interferon gene response is increased in early and established rheumatoid arthritis and correlates with autoantibody production. Front Immunol 2017;8:285. 28373872 10.3389/fimmu.2017.00285 PMC5357778

[29] Wright HL , Thomas HB , Moots RJ , Edwards SW . Interferon gene expression signature in rheumatoid arthritis neutrophils correlates with a good response to TNFi therapy. Rheumatology (Oxford) 2015;54:188–93. 25125592 10.1093/rheumatology/keu299

[30] Vosslamber S , Raterman HG , van der Pouw Kraan TC , Schreurs MW , von Blomberg BM , Nurmohamed MT , et al. Pharmacological induction of interferon type I activity following treatment with rituximab determines clinical response in rheumatoid arthritis. Ann Rheum Dis 2011;70:1153–9. 21444302 10.1136/ard.2010.147199

[31] Sellam J , Marion‐Thore S , Dumont F , Jacques S , Garchon HJ , Rouanet S , et al. Use of whole‐blood transcriptomic profiling to highlight several pathophysiologic pathways associated with response to rituximab in patients with rheumatoid arthritis: data from a randomized, controlled, open‐label trial. Arthritis Rheumatol 2014;66:2015–25. 24756903 10.1002/art.38671

[32] Sanayama Y , Ikeda K , Saito Y , Kagami S , Yamagata M , Furuta S , et al. Prediction of therapeutic responses to tocilizumab in patients with rheumatoid arthritis: biomarkers identified by analysis of gene expression in peripheral blood mononuclear cells using genome‐wide DNA microarray. Arthritis Rheumatol 2014;66:1421–31. 24591094 10.1002/art.38400

[33] Raterman HG , Vosslamber S , de Ridder S , Nurmohamed MT , Lems WF , Boers M , et al. Interferon type I signature may predict non response upon rituximab in rheumatoid arthritis patients. Arthritis Res Ther 2012;14:R95. 22540992 10.1186/ar3819 PMC3446469

[34] Mesko B , Poliska S , Váncsa A , Szekanecz Z , Palatka K , Hollo Z , et al. Peripheral blood derived gene panels predict response to infliximab in rheumatoid arthritis and Crohn's disease. Genome Med 2013;5:59. 23809696 10.1186/gm463 PMC4064310

[35] Curtis JR , Wallenstein G , Takiya L , Gruben D , Chen C , Shan Y , et al. Patterns of methotrexate use and discontinuation in a U.S. rheumatoid arthritis registry [abstract]. Arthritis Rheumatol 2017;69 Suppl 10. URL: http://acrabstracts.org/abstract/patterns-of-methotrexate-use-and-discontinuation-in-a-u-s-rheumatoid-arthritis-registry/.

[36] Prevoo ML , van't Hof MA , Kuper HH , van Leeuwen MA , van de Putte LB , van Riel PL . Modified disease activity scores that include twenty‐eight–joint counts: development and validation in a prospective longitudinal study of patients with rheumatoid arthritis. Arthritis Rheum 1995;38:44–8. 7818570 10.1002/art.1780380107

[37] Cordingley L , Prajapati R , Plant D , Maskell D , Morgan C , Ali FR , et al. Impact of psychological factors on subjective disease activity assessments in patients with severe rheumatoid arthritis. Arthritis Care Res (Hoboken) 2014;66:861–8. 24339425 10.1002/acr.22249 PMC4153952

[38] Ton E , Bakker MF , Verstappen SM , Ter Borg EJ , van Albada‐Kuipers IA , Schenk Y , et al. Look beyond the Disease Activity Score of 28 Joints (DAS28): tender points influence the DAS28 in patients with rheumatoid arthritis. J Rheumatol 2012;39:22–7. 22002014 10.3899/jrheum.110072

