# Supplementary material for: Profiling of Gene Expression Biomarkers as a Classifier of Methotrexate Nonresponse in Patients With Rheumatoid Arthritis
Source: Arthritis Rheumatol. 2019 Mar 19;71(5):678–84. doi: 10.1002/art.40810 (PMC9328381; doi:10.1002/art.40810)
Supplement: Supplementary file 1 — Supplementary Figures [file ART-71-678-s001.pdf]

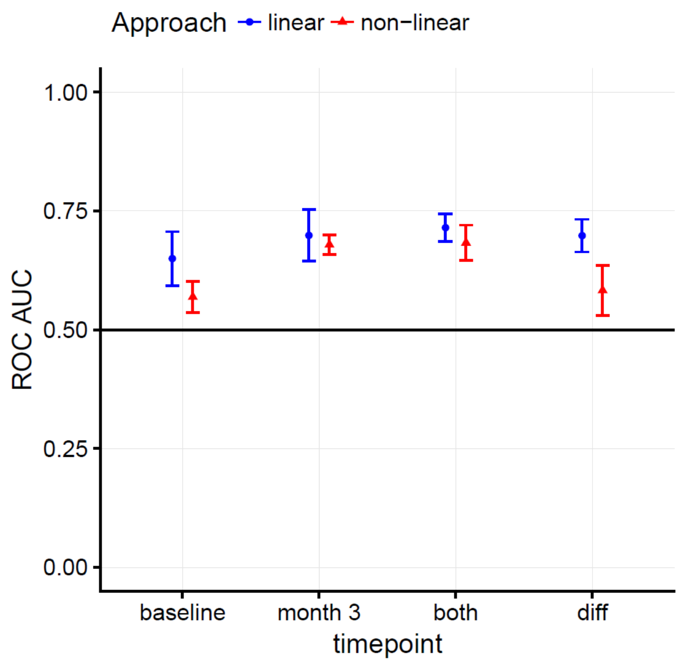

Supplementary Figure 1: Average ROC AUCs across the cross-validation runs from models based on clinical data. Results are shown for baseline; 3-months on drug, both time-points combined and for the difference between time-points.

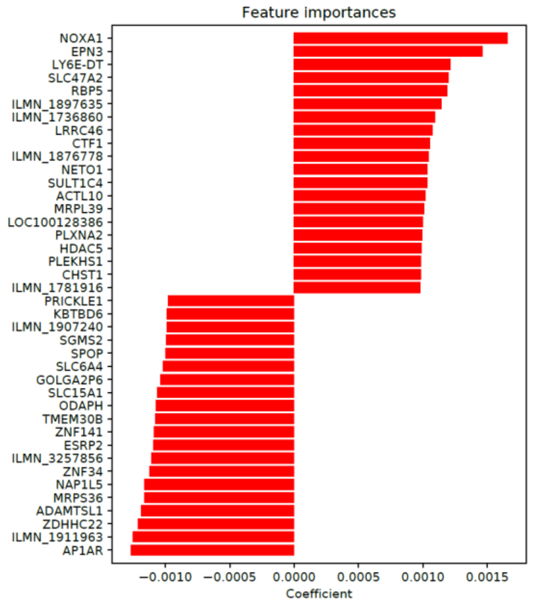

Supplementary Figure 2: Coefficients in linear response models of gene expression ratio data (only the top 20 and bottom 20 coefficients are shown for illustration). Gene symbol or, in absence of gene symbol, illumina probe ID are presented on the y-axis; coefficient magnitude is represented by the size of the red bars.

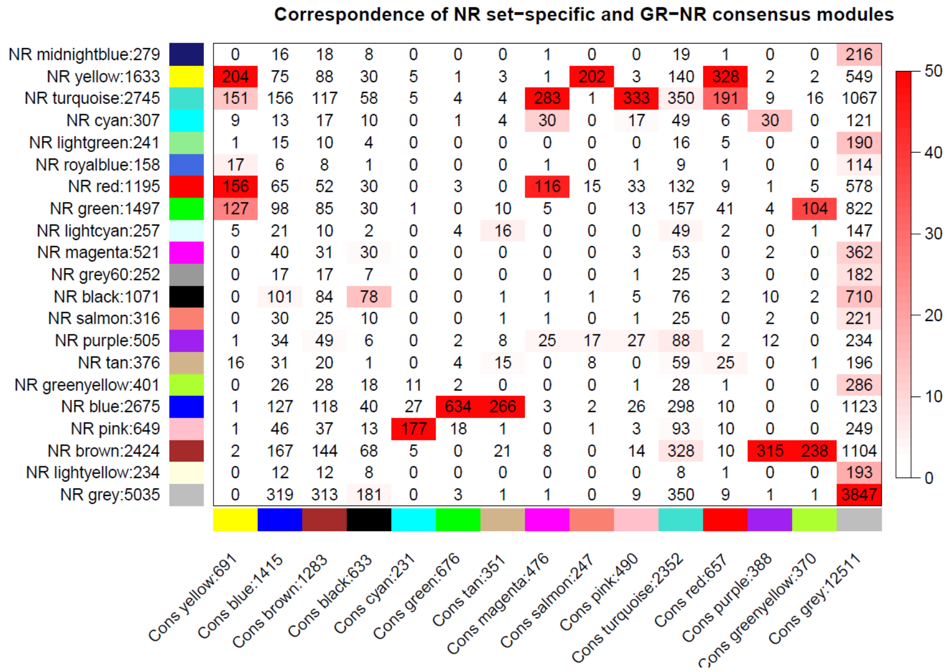

Supplementary Figure 3: Correspondence of the MTX non-responder (NR) set-specific and the good-responder-NR consensus modules in pre-treatment samples. Each row of the table corresponds to one NR module (labelled by colour as well as text), and each column corresponds to one consensus module, i.e. modular structure in all pre-treatment samples. Numbers in the table indicate gene counts in the intersection of the corresponding modules. Colouring of the table encodes log(p), with p being the hypergeometric test p-value for the overlap of the two modules. The stronger the red colour, the more significant the overlap is. The table indicates that most NR set-specific modules have a consensus counterpart i.e. the module structure is similar between NR and GR. Interestingly, there are nine NR modules (labelled by midnight blue, Light green, royal blue, magenta, grey60, black, salmon, green-yellow and light-yellow colours) that have no direct consensus counterpart; over 65% (arbitrary threshold) of genes in the nine NR modules are labelled grey, that is unassigned, in the consensus network.

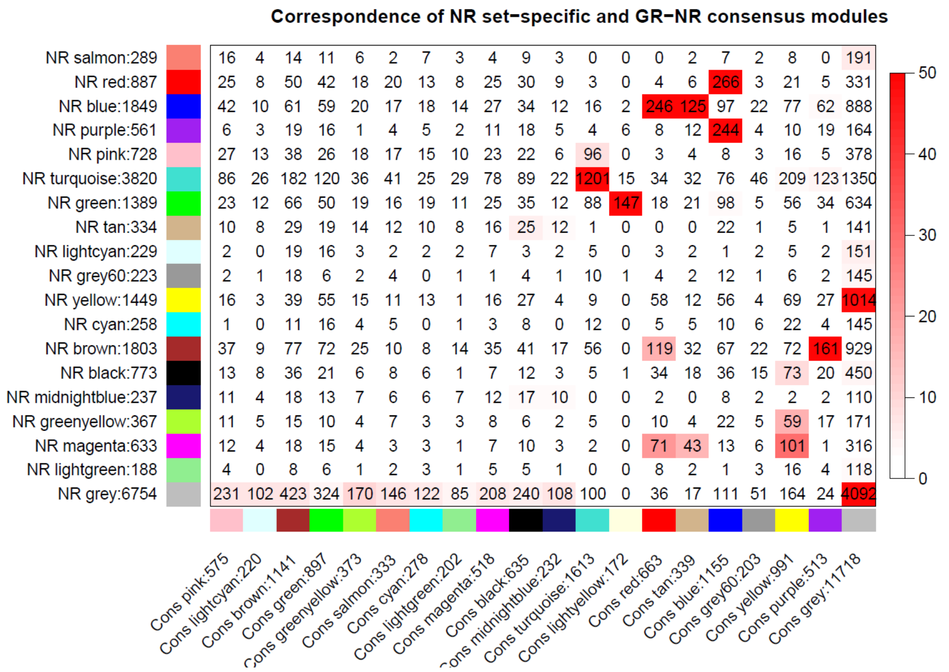

Supplementary Figure 4: Correspondence of the MTX non-responder (NR) set-specific and the GR-NR consensus modules in 4-week samples. Each row of the table corresponds to one NR module (labelled by colour as well as text), and each column corresponds to one consensus module, i.e. modular structure in all 4-week samples. Numbers in the table indicate gene counts in the intersection of the corresponding modules. Colouring of the table encodes log(p), with p being the hypergeometric test p-value for the overlap of the two modules. The stronger the red colour, the more significant the overlap is. The table indicates that many non-responder specific modules have a consensus counterpart i.e. the module structure is similar between NR and GR. Interestingly the salmon, light cyan, grey60 and yellow modules have no direct consensus counterpart; over 65% (arbitrary threshold) of genes in these NR modules are labelled grey, which is unassigned, in the consensus network.

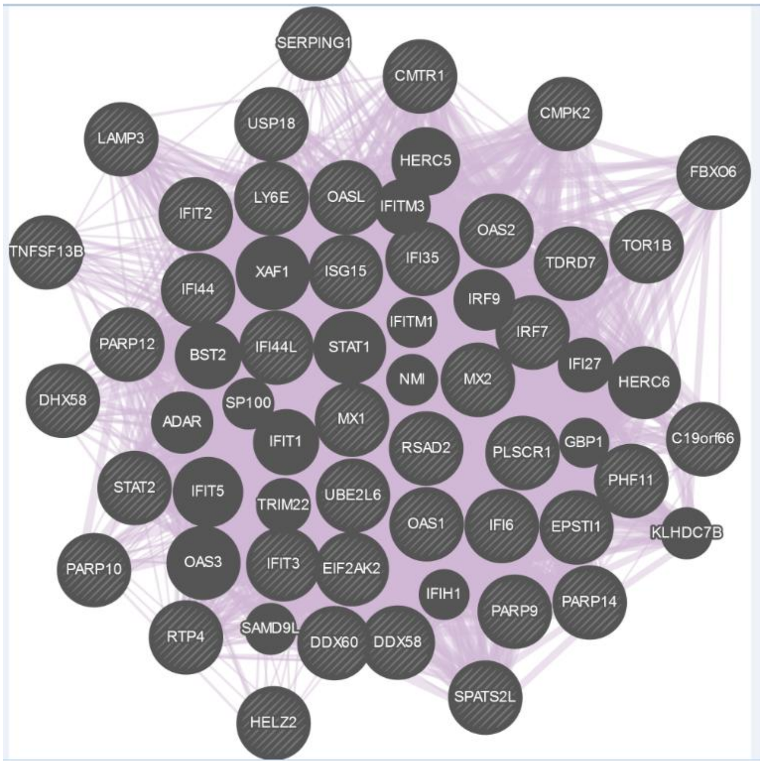

Supplementary figure 5: The genes from within the light green module identified at pre-treatment in non-responder patients were queried for prior evidence of co-expression against Gene Expression Omnibus data. Of the genes queried (identified by grey stripes) 96% had prior evidence of co-expression indicated by the connecting lines between genes.

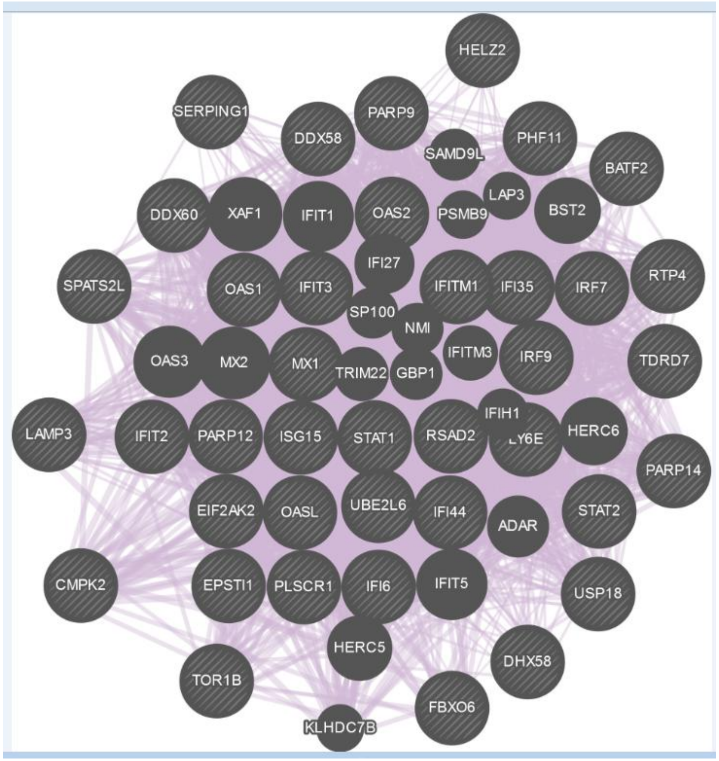

Supplementary figure 6: The genes from within the light cyan module identified at 4-weeks in non-responder patients were queried for prior evidence of co-expression against Gene Expression Omnibus data. Of the genes queried (identified by grey stripes) 93% had prior evidence of co-expression indicated by the connecting lines between genes.

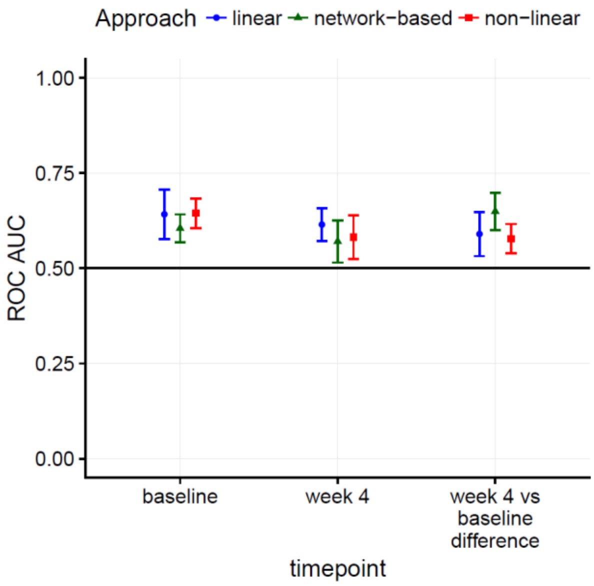

Supplementary Figure 7: Average ROC AUCs across the cross-validation runs from models based on interferon pathway gene transcripts. Results are shown for baseline (pre-treatment), after 4-weeks on drug and using the ratio of gene expression between 4-weeks and pre-treatment.

RAMS Co-investigators

**Trust Name**	**Investigator Name**
Manchester University Hospitals NHS Foundation Trust	Professor Kimme Hyrich
Norfolk and Norwich University Hospitals NHS Foundation Trust	Dr. Tarnya Marshall
East Cheshire NHS Trust	Dr. Susan Knight
East Lancashire Hospitals NHS Trust	Dr. Lee-Suan Teh
Tameside and Glossop Integrated Care NHS Foundation Trust	Dr. Dipak Roy
Salford Royal NHS Foundation Trust	Dr. Hector Chinoy
South Warwickshire NHS Foundation Trust	Dr. Christopher Marguerie
University Hospitals of Morecambe Bay NHS Foundation Trust	Dr. Marwan Bukhari
King's College Hospital NHS Foundation Trust	Dr. James Galloway
Royal Cornwall Hospitals NHS Trust	Dr. Martin Davis
The Rotherham NHS Foundation Trust	Dr. Gillian Smith
York Teaching Hospital NHS Foundation Trust	Dr. Michael Green
Harrogate and District NHS Foundation Trust	Dr. Andrew Gough
The Royal Bournemouth and Christchurch Hospitals NHS Foundation Trust	Dr. Brian Quilty
Torbay and South Devon NHS Foundation Trust	Dr. Nick Viner
Salisbury NHS Foundation Trust	Dr. Richard Smith
Barnsley Hospital NHS Foundation Trust	Dr. Ade Adebajo
The Robert Jones and Agnes Hunt Orthopaedic Hospital NHS Foundation Trust	Dr. Roshan Amarasena
Southend University Hospital NHS Foundation Trust	Professor Bhaskar Dasgupta
Staffordshire and Stoke on Trent Partnership NHS Trust	Dr. Samantha Hider
Lewisham and Greenwich NHS Trust	Dr. Louise Pollard
University Hospitals of Leicester NHS Trust	Dr. Waji Hassan
The Ipswich Hospital NHS Trust	Dr. Suzanne Lane
Gateshead Health NHS Foundation Trust	Dr. Vadivelu Saravanan
Chelsea and Westminster Hospital NHS Foundation Trust	Professor Margaret Callan
Croydon Health Services NHS Trust	Dr. Sarah Levy
Plymouth Hospitals NHS Trust	Dr. Nick Viner
Lancashire Care NHS Foundation Trust	Dr. Lizzy Macphie
The Newcastle upon Tyne Hospitals NHS Foundation Trust	Dr. Martin Lee
Princess Alexandra Hospital NHS Trust	Dr. Khalid Ahmed
South London Healthcare NHS Trust	Dr. Catherine Mathews
South London Healthcare NHS Trust (no longer exists)	Dr. Catherine Mathews
South London Healthcare NHS Trust (no longer exists)	Dr. Catherine Mathews
University Hospital of South Manchester NHS Foundation Trust	Dr. Paul Sanders
Hampshire Hospitals NHS Foundation Trust	Dr. Emma Williams
Central Manchester University Hospitals NHS Foundation Trust	Dr. Frank McKenna
Wrightington, Wigan and Leigh NHS Foundation Trust	Dr. Easwaradhas Gladston Chelliah
The Pennine Acute Hospitals NHS Trust	Dr. Sophia Naz

BRAGGSS Co-investigators

Barts Health NHS Trust	Prof C Pitzalis
Basingstoke & North Hampshire NHS Foundation Trust	Dr E Williams, Dr R K Moitra, Dr D J Shawe
Burton Hospitals NHS Foundation Trust	Dr R Laxminarayan
Central Manchester University Hospitals NHS Foundation Trust	Prof I Bruce, Prof A Barton, Dr R Gorodkin, Dr P Ho, Dr K Hyrich, Dr F McKenna
Chesterfield Royal Hospital NHS Foundation Trust	Dr K Fairburn
Countess of Chester Hospital NHS Foundation Trust	Dr J Nixon, Dr T Barnes, Dr M Hui
City Hospitals Sunderland NHS Foundation Trust	Dr D Coady, Dr D Wright, Dr C Morley, Dr G Raftery, Dr C Bracewell
County Durham and Darlington NHS Foundation Trust	Dr R Reece, Dr. D. Armstrong, Dr. A J Chuck, Dr. S Hailwood, Dr N Kumar, Dr D Ashok
Derby Hospitals NHS Foundation Trust	Dr. S C O'Reilly, Dr T Ding, Dr. L J Badcock, Dr. C M Deighton , Dr N Raj, Dr. M R Regan, Dr. G D Summers, Dr. R A Williams
Gateshead Health NHS Foundation Trust	Dr. C A Kelly, Dr. J Hamilton, Dr. C R Heycock, Dr V Saravanan
Harrogate & District NHS Foundation Trust	Dr M Green, Dr A Gough, Dr C Lawson
Kettering General Hospital NHS Foundation Trust	Dr A Kuttikat, Dr D Parthajit, Dr E Borbas, Dr T Wazir
Leeds Teaching Hospitals NHS Trust	Prof. P Emery, Dr. S. Bingham, Prof. A. Morgan, Prof H A Bird, Prof P G Conaghan, Dr C T Pease, Dr R J Wakefield, Prof. M Buch, Dr S Dass
Northumbria Healthcare NHS Foundation Trust	Dr F N Birrell, Dr P R Crook
Northern Lincolnshire and Goole Hospitals NHS Foundation Trust	Dr B Szebenyi, Dr D Bates, Dr D James, Dr T Gillott, Dr A Alvi, C Grey, J Browning
Nottingham University Hospitals NHS Trust	Dr. J F McHale, Dr. I C Gaywood, Dr. A C Jones, Dr. P Lanyon, Dr. I Pande, Prof. M Doherty, Dr. A Gupta, Dr. P A Courtney, Dr A Srikanth, Dr A Abhishek
Pennine Acute Hospitals NHS Trust	Dr L Das, Dr. M Pattrick, Dr. H N Snowden, Dr A P Bowden, Dr E E Smith, Dr P Klimiuk, Dr D J Speden, Dr L Das
Portsmouth Hospitals NHS Trust	Dr. J M Ledingham , Dr. R G Hull, Dr. F McCrae, Dr. A Cooper, Dr S A Young Min, Dr Wong, Dr Shaban
Royal Cornwall Hospitals NHS Trust	Prof A D Woolf, Dr M Davis, Dr D Hutchinson, Dr A Endean
Royal Liverpool and Broadgreen University Hospitals NHS Trust	Dr D Mewar, Dr E J Tunn, Dr K Nelson, Dr T D Kennedy, Dr C Dubois
Royal National Hospital for Rheumatic Diseases NHS Foundation Trust	Dr J Pauling, Dr E Korendowych, Dr T Jenkinson, Dr R Sengupta, Dr A Bhalla, Prof N McHugh, Dr W Tillett, Dr T Ahmed
Salford Royal NHS Foundation Trust	Dr. H Chinoy, Prof T O'Neil, Prof A Herrick, Prof A Jones, Dr R Cooper, Dr W Dixon, Dr B Harrison
Sheffield Teaching Hospitals NHS Foundation Trust	Dr. M Akil, Dr. S Till, Dr L Dunkley, Dr R Tattersall, Dr R Kilding, Dr T Tait, Dr J Maxwell, Dr K-P Kuet
South Tees Hospitals NHS Foundation Trust	Dr. M J Plant, Dr. F Clarke, Dr. J N Fordham, Dr S Tuck, Dr S K Pathare, Dr A Paul
South Warwickshire General Hospital NHS Trust	Dr. C P Marguerie, Dr S P Rigby, Dr N Dunn
Staffordshire & Stoke-on-Trent Partnership NHS Trust	Dr S Hider, Dr A Menon, Dr C Dowson, Dr S Dutta, Dr S Kamath, Dr J Packham, Dr S Price, Dr E Roddy, Dr Z Paskins, Professor A. Hassell
Stockport NHS Foundation Trust	Dr A Ismail, Dr C Filer
St Helens and Knowsley Hospitals NHS Trust	Dr. R Abernethy, Dr A R Clewes, Dr. J K Dawson
The Dudley Group of Hospitals NHS Foundation Trust	Prof G Kitas, Dr N Erb, Dr R Klocke, Dr A J Whallett, Dr K Douglas, Dr A Pace, Dr R Sandhu, Dr H John
The Ipswich Hospital NHS Trust	Dr S Lane
The Newcastle upon Tyne Hospitals NHS Foundation Trust	Prof. J D Isaacs, Prof. H Foster, Dr. B Griffiths, Dr. I Griffiths, Dr L Kay, Dr W-F Ng, Dr. P N Platt, Dr. D J Walker, Dr P Peterson, Dr A Lorenzi, Dr M Friswell, Dr B Thompson, Dr M Lee, Dr A Pratt
The Royal Wolverhampton Hospitals NHS Trust	Dr. D Mulherin, Dr. S V Chalam, , Dr. T Price, Dr. T Sheeran, Dr S Venkatachalam, Dr S Baskar, Dr Sabrina Raizada
University Hospital Birmingham NHS Foundation Trust	Dr A Filer, Dr. Bowman, Dr. P Jobanputra Dr. E C Rankin
University Hospitals of Coventry and Warwickshire NHS Trust	Dr S Dubey, Dr. K Chaudhuri, Dr A Price-Forbes, Dr J Ravindran
University Hospitals of Leicester NHS Trust	Dr A Moorthy, Dr P Sheldon, Dr W Hassan, Dr J Francis, Dr A Kinder, Dr R Neame
University Hospitals of Morcambe Bay NHS Trust	Dr. M Bukhari, Dr L Ottewell, Dr Palkonyai, Dr M Bukhari
West Suffolk Hospitals NHS Trust	Dr D T O'Reilly, Dr V Rajagopal
Wrightington, Wigan and Leigh Hospitals NHS Foundation Trust	Dr E Gladston Chelliah
York Teaching Hospitals NHS Foundation Trust	Dr M Green, Dr M Quinn, Dr A Isdale, Dr A Brown, Dr B Saleem, Dr Z Al-Saffar, Dr G Koduri
