# Supplementary material for: Profiling of Gene Expression Biomarkers as a Classifier of Methotrexate Nonresponse in Patients With Rheumatoid Arthritis
Source: Arthritis Rheumatol. 2019 Mar 19;71(5):678–84. doi: 10.1002/art.40810 (PMC9328381; doi:10.1002/art.40810)
Supplement: Supplementary file 2 — Supplementary Tables [file ART-71-678-s002.pdf]

Supplementary Table1: Functional analysis of gene lists from modules. The Top term from each module analysed is reported.

Module	Responder status	Time-point	GO BP ID	count	Size	Pvalue	Term
Black	NR	PT	GO:0014042	2	4	6.20E-04	positive regulation of neuron maturation
Greenyellow	NR	PT	GO:0045664	9	439	5.28E-05	regulation of neuron differentiation
Grey60	NR	PT	GO:0007596	19	446	4.91E-17	blood coagulation
Lightgreen	NR	PT	GO:0034340	15	62	2.76E-25	response to type I interferon
Lightyellow	NR	PT	GO:0035020	2	10	3.23E-04	regulation of Rac protein signal transduction
Magenta	NR	PT	GO:0051453	4	38	2.89E-05	regulation of intracellular pH
Midnightblue	NR	PT	GO:0019226	4	54	4.01E-05	transmission of nerve impulse
Salmon	NR	PT	GO:0001545	1	1	2.83E-03	primary ovarian follicle growth
grey60	NR	4WK	GO:0007596	17	446	6.13E-14	blood coagulation
Lightcyan	NR	4WK	GO:0060337	16	58	4.94E-28	type I interferon signaling pathway
Salmon	NR	4WK	GO:0000730	2	6	1.70E-04	DNA recombinase assembly
Yellow	NR	4WK	GO:0006955	47	1207	4.96E-06	immune response

NR=non-responders, PT=pre-treatment, 4WK=4-weeks, GO BP ID=gene ontology biologicalprocess identifier, count=number of genes in list, Size=number of genes in universe (arrayed genes), Pvalue=hypergeometric p-value

Supplementary Table2: Annotation of lightgreen module hub genes.

EntrezID	Symbol	Names	GO_term_BP
55337	C19orf66	chromosome 19 open reading frame 66	
129607	CMPK2	cytidine/uridine monophosphate kinase 2	
23070	CMTR1	cap methyltransferase 1	
23586	DDX58	DEXD/H-box helicase 58	
55601	DDX60	DEXD/H-box helicase 60	
79132	DHX58	DEXH-box helicase 58	
5610	EIF2AK2	eukaryotic translation initiation factor 2 alpha kinase 2	
94240	EPSTI1	epithelial stromal interaction 1	
26270	FBXO6	F-box protein 6	
85441	HELZ2	helicase with zinc finger 2	
3430	IFI35	interferon induced protein 35	response to type I interferon
10561	IFI44	interferon induced protein 44	response to type I interferon
10964	IFI44L	interferon induced protein 44 like	response to type I interferon
2537	IFI6	interferon alpha inducible protein 6	response to type I interferon
3433	IFIT2	interferon induced protein with tetratricopeptide repeats 2	response to type I interferon
3437	IFIT3	interferon induced protein with tetratricopeptide repeats 3	response to type I interferon
3665	IRF7	interferon regulatory factor 7	response to type I interferon
9636	ISG15	ISG15 ubiquitin-like modifier	response to type I interferon
27074	LAMP3	lysosomal associated membrane protein 3	
4061	LY6E	lymphocyte antigen 6 complex, locus E	
4599	MX1	MX dynamin like GTPase 1	response to type I interferon
4938	OAS1	2'-5'-oligoadenylate synthetase 1	response to type I interferon
4939	OAS2	2'-5'-oligoadenylate synthetase 2	response to type I interferon
8638	OASL	2'-5'-oligoadenylate synthetase like	response to type I interferon
84875	PARP10	poly(ADP-ribose) polymerase family member 10	
64761	PARP12	poly(ADP-ribose) polymerase family member 12	
54625	PARP14	poly(ADP-ribose) polymerase family member 14	
83666	PARP9	poly(ADP-ribose) polymerase family member 9	
51131	PHF11	PHD finger protein 11	
5359	PLSCR1	phospholipid scramblase 1	

91543	RSAD2	radical S-adenosyl methionine domain containing 2	response to type I interferon
64108	RTP4	receptor transporter protein 4	
710	SERPING1	serpin family G member 1	
26010	SPATS2L	spermatogenesis associated serine rich 2 like	
6773	STAT2	signal transducer and activator of transcription 2	response to type I interferon
23424	TDRD7	tudor domain containing 7	
10673	TNFSF13B	tumor necrosis factor superfamily member 13b	
27348	TOR1B	torsin family 1 member B	
9246	UBE2L6	ubiquitin conjugating enzyme E2 L6	
11274	USP18	ubiquitin specific peptidase 18	response to type I interferon

GO_term_BP = gene ontology biological process

Supplementary Table3: Annotation of lightcyan module hub genes.

EntrezID	Symbol	Name	GO_term_BP
116071	BATF2	basic leucine zipper ATF-like transcription factor 2	
129607	CMPK2	cytidine/uridine monophosphate kinase 2	
23586	DDX58	DEXD/H-box helicase 58	
55601	DDX60	DEXD/H-box helicase 60	
79132	DHX58	DEXH-box helicase 58	
5610	EIF2AK2	eukaryotic translation initiation factor 2 alpha kinase 2	
94240	EPSTI1	epithelial stromal interaction 1	
26270	FBXO6	F-box protein 6	
85441	HELZ2	helicase with zinc finger 2	
3430	IFI35	interferon induced protein 35	type I interferon signaling pathway
10561	IFI44	interferon induced protein 44	
10964	IFI44L	interferon induced protein 44 like	
2537	IFI6	interferon alpha inducible protein 6	type I interferon signaling pathway
3433	IFIT2	interferon induced protein with tetratricopeptide repeats 2	type I interferon signaling pathway
3437	IFIT3	interferon induced protein with tetratricopeptide repeats 3	type I interferon signaling pathway
8519	IFITM1	interferon induced transmembrane protein 1	type I interferon signaling pathway
3665	IRF7	interferon regulatory factor 7	type I interferon signaling pathway
10379	IRF9	interferon regulatory factor 9	type I interferon signaling pathway
9636	ISG15	ISG15 ubiquitin-like modifier	type I interferon signaling pathway
27074	LAMP3	lysosomal associated membrane protein 3	
4061	LY6E	lymphocyte antigen 6 complex, locus E	
4599	MX1	MX dynamin like GTPase 1	type I interferon signaling pathway
4938	OAS1	2'-5'-oligoadenylate synthetase 1	type I interferon signaling pathway
4939	OAS2	2'-5'-oligoadenylate synthetase 2	type I interferon signaling pathway
8638	OASL	2'-5'-oligoadenylate synthetase like	type I interferon signaling pathway
64761	PARP12	poly(ADP-ribose) polymerase family member 12	
54625	PARP14	poly(ADP-ribose) polymerase family member 14	
83666	PARP9	poly(ADP-ribose) polymerase family member 9	
51131	PHF11	PHD finger protein 11	
5359	PLSCR1	phospholipid scramblase 1	
91543	RSAD2	radical S-adenosyl methionine domain containing 2	type I interferon signaling pathway
64108	RTP4	receptor transporter protein 4	
710	SERPING1	serpin family G member 1	
3431	SP110	SP110 nuclear body protein	
26010	SPATS2L	spermatogenesis associated serine rich 2 like	
6772	STAT1	signal transducer and activator of transcription 1	type I interferon signaling pathway

6773	STAT2	signal transducer and activator of transcription 2	type I interferon signaling pathway
23424	TDRD7	tudor domain containing 7	
27348	TOR1B	torsin family 1 member B	
9246	UBE2L6	ubiquitin conjugating enzyme E2 L6	
11274	USP18	ubiquitin specific peptidase 18	type I interferon signaling pathway

GO_term_BP = gene ontology biological process
